# Deep learning for differential diagnosis of parotid tumors based on 2.5D magnetic resonance imaging

**DOI:** 10.1080/07853890.2025.2520401

**Published:** 2025-06-18

**Authors:** Wenfeng Mai, Xiaole Fan, Lingtao Zhang, Jian Li, Liting Chen, Xiaoyu Hua, Dong Zhang, Hengguo Li, Minxiang Cai, Changzheng Shi, Xiangning Liu

**Affiliations:** a Medical Imaging Center, The First Affiliated Hospital of Jinan University, Guangzhou, China; b Department of Ultrasound, The First Affiliated Hospital of Jinan University, Guangzhou, China; c Department of Radiology, The First Affiliated Hospital, Jiangxi Medical College, Nanchang University, Nanchang, China; d Clinical Research Platform for Interdiscipline of Stomatology, School of Stomatology, The First Affiliated Hospital of Jinan University, Jinan University, Guangzhou, China

**Keywords:** Magnetic resonance imaging, parotid tumors, deep learning, 2.5D imaging

## Abstract

**Purpose:**

Accurate preoperative diagnosis of parotid gland tumors (PGTs) is crucial for surgical planning since malignant tumors require more extensive excision. Though fine-needle aspiration biopsy is the diagnostic gold standard, its sensitivity in detecting malignancies is limited. While Deep learning (DL) models based on magnetic resonance imaging (MRI) are common in medicine, they are less studied for parotid gland tumors. This study used a 2.5D imaging approach (Incorporating Inter-Slice Information) to train a DL model to differentiate between benign and malignant PGTs.

**Methods:**

This retrospective study included 122 parotid tumor patients, using MRI and clinical features to build predictive models. In the traditional model, univariate analysis identified statistically significant features, which were then used in multivariate logistic regression to determine independent predictors. The model was built using four-fold cross-validation. The deep learning model was trained using 2D and 2.5D imaging approaches, with a transformer-based architecture employed for transfer learning. The model’s performance was evaluated using the area under the receiver operating characteristic curve (AUC) and confusion matrix metrics.

**Results:**

In the traditional model, boundary and peritumoral invasion were identified as independent predictors for PGTs, and the model was constructed based on these features. The model achieved an AUC of 0.79 but demonstrated low sensitivity (0.54). In contrast, the DL model based on 2.5D T2 fat-suppressed images showed superior performance, with an AUC of 0.86 and a sensitivity of 0.78.

**Conclusion:**

The 2.5D imaging technique, when integrated with a transformer-based transfer learning model, demonstrates significant efficacy in differentiating between PGTs.

## Introduction

Salivary gland tumors (SGTs) account for 2–6.5% of head and neck tumors, with PGTs being the most common. About 80% of PGTs are benign, mainly pleomorphic adenomas and Warthin’s tumors [1–5]. Malignant parotid gland tumors (MPGTs), including mucoepidermoid carcinoma (MEC) and lymphoma, are rarer. SGT distribution varies by gender: squamous cell carcinoma is more common in males, while acinic cell carcinoma and adenoid cystic carcinoma are more prevalent in females [6,7]. MEC is more common in women within 50 years of age and in men over 50 [6]. The rising incidence of SGTs underscores the need for research.

Diagnosing PGTs is challenging due to their morphological diversity and overlapping features, complicating differentiation between benign and malignant forms [8]. Fine needle aspiration biopsy, the gold standard, can be limited by insufficient tissue, tumor size, and location, leading to misdiagnosis [9,10]. Magnetic resonance imaging, crucial for tumor localization and distinguishing PGTs, varies in effectiveness depending on radiologist expertise and similar imaging characteristics [2,11–15]. Combining pathology with advanced magnetic resonance imaging (MRI) techniques is essential for improving diagnostic accuracy and guiding appropriate treatment, whether conservative surgery or more extensive interventions [16–19].

Recent advancements in DL have shown the potential of 2.5D strategies, utilizing multi-view fusion, inter-slice information, or a blend of 2D and 3D features for lesion analysis [20,21]. While 2.5D DL models based on computed tomography images have been successfully applied to segment lesions in maxillary sinuses and nasopharyngeal carcinoma, as well as to predict human papillomavirus infection in advanced cases [22–24], their application in PGTs remains in the exploratory stage. In this study, we integrated inter-slice information within a 2.5D framework, combined with a DL model enhanced by attention mechanisms, to distinguish between benign and malignant PGTs using conventional MRI, thereby supporting clinical decision-making.

## Materials and methods

### Ethical statement

This study was conducted in accordance with the ethical standards of the Declaration of Helsinki and was approved by the local ethics review committee of the First Affiliated Hospital of Jinan University (KY-2023-360). The committee granted a waiver of informed consent under specific conditions, considering the retrospective nature of the study and the use of anonymized patient data.

### Patient cohorts

This retrospective study consecutively collected clinical records, radiological data, and pathology reports of patients with PGTs from the First Affiliated Hospital of Jinan University (City A Hospital, January 2015 to May 2023) and the First Affiliated Hospital of Nanchang University (City B Hospital, January 2020 to May 2023). Clinical data were sourced from the hospital’s clinical management system, and MRI data were retrieved from the Picture Archiving and Communication System. The definitive postoperative pathological diagnosis, considered the gold standard for this study, was confirmed through the patient’s pathology report.

The inclusion and exclusion criteria for patient selection were as follows:

#### Inclusion criteria

1. Complete clinical and radiological data;
2. Tumors distinctly located in the parotid gland;
3. MRI conducted within 14 days prior to surgery;
4. A definitive pathological diagnosis.

#### Exclusion criteria

1. Recurrent tumor;
2. Incomplete or poor-quality MRI data;
3. Tumors smaller than 5 mm, making accurate delineation in the region of interest challenging (ROI).

### Image acquisition

Axial T1-weighted images (T1WI) and fat-saturated T2-weighted images (T2WI-FS) were acquired from patients using GE 3.0 T Discovery MR750 and GE 1.5 T OPTIMA superconducting MR scanners at City A Hospital, and a SIEMENS 3.0 T superconducting MR scanner at City B Hospital. Scanning parameters were detailed in Table 1. Due to consistent availability and superior lesion visualization, T2WI-FS was selected over standard T2-weighted images. Consequently, T2WI-FS was selected for analysis to ensure uniformity across the datasets from both centers.

**Table 1.** Detailed scanning parameters for each sequence.

	Hospital A		Hospital B	
Sequence	Axial T1WI	Axial T2WI-FS	Axial T1WI	Axial T2WI-FS
TR (ms)	517-756	4017-4443	500-710	5000-7690
TE(ms)	11-14	80-88	9-13	60-102
Acquisition matrix	288 × 224	288 × 256	320 × 200	320 × 224
Slice thickness	4	4	5	5
Field of view	240 × 288	240 × 288	240 × 288	240 × 288
NEX	1	1	1	1

### Medical imaging characteristics assessment

Two radiologists independently assessed the medical imaging characteristics, one with over 15 years and the other with over 5 years of experience in head and neck imaging. The evaluated characteristics included tumor size, shape (regular or irregular), boundary clarity (clear or blurred), invasion of surrounding tissues (positive or negative), lymph node status (no enlargement or enlargement), location (superficial lobe, deep lobe, or trans-lobar growth), and distribution (right or left side). Any discrepancies in their assessments were resolved through discussion to reach a consensus, ensuring consistency and accuracy in data collection.

### ROI delineation and image preparation

Tumor ROIs were delineated using ITK-SNAP software (version 4.0.1) by two radiologists with five years of experience [25]. The regions were then expanded by 15 pixels from their longest diameter to include surrounding tissue, providing a more comprehensive context for subsequent 2D and 2.5D images analysis.

The 2.5D images were generated by stacking three consecutive grayscale MRI slices [20,21], enabling the capture of inter-slice information, while 2D images were based on single-slice grayscale images. All tumor slices were utilized for model training and validation, with the independent test set evaluated using the largest tumor cross-sections.

### Model training

A traditional model was constructed using data from City A Hospital with 4-fold cross-validation to ensure robustness. Initially, univariate analysis was conducted to identify clinical and radiological characteristics with statistical significance. Subsequently, multivariate regression analysis was employed to determine the independent predictors for distinguishing between benign and malignant PGTs, forming the basis for the predictive model. The model’s performance was evaluated using metrics such as average AUC, sensitivity, specificity, and F1 score, ensuring accuracy and stability in diagnosis. In deep learning and machine learning models, the F1 Score is a metric used to evaluate the performance of a classification model. If the F1 Score is high and stable, it indicates that the model performs in terms of accuracy and comprehensiveness better in recognizing positive class samples.

**Figure 1.**
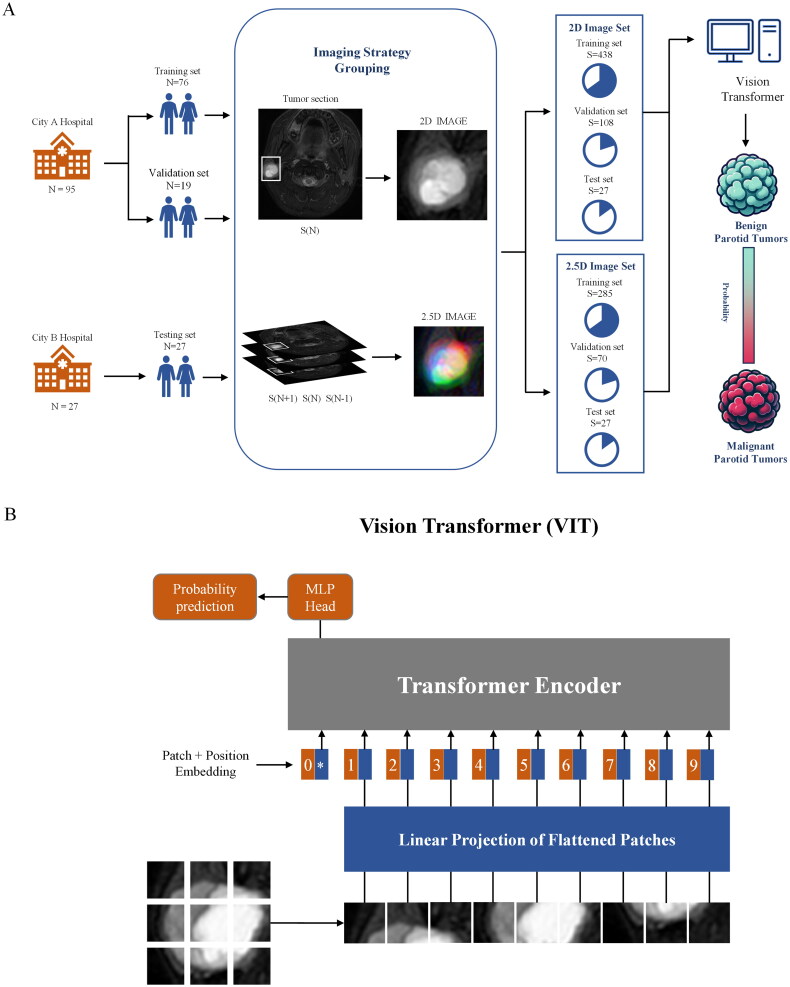
Imaging strategy and model workflow. (A) Illustration of the imaging strategy and data grouping process, showing the division of tumor slices into 2D and 2.5D image sets from two hospitals. (B) Workflow of the deep learning model using the vision transformer, where input images are divided into patches, projected linearly, and processed through the transformer encoder to predict tumor probability, differentiating between PGTs.

The DL model’s cohort division and a brief overview of the Vision Transformer (ViT-B/16) architecture are illustrated in Figure 1(A,B). The dataset from the First Affiliated Hospital of Jinan University was further divided into a training set and a validation set for DL model training and hyperparameter optimization, while the dataset from the First Affiliated Hospital of Nanchang University was employed as an independent test set for model evaluation. The patient cohort was divided into training and internal validation sets in an 8:2 ratio. A pre-trained Vision Transformer model was utilized for image classification. Data preprocessing included random cropping to 224 × 224 pixels, random horizontal flipping, and normalization (mean and standard deviation set to 0.5). Model training was performed using the SGD optimizer with an initial learning rate of 0.42, momentum of 0.9, and a weight decay of 5E-5, with the learning rate adjusted via a cosine annealing schedule. Training was conducted with a batch size of 22 over 50 epochs, leveraging GPU acceleration. Selective layer freezing was applied to expedite the training process. Both 2.5D and 2D images were employed, with data augmentation techniques utilized to further mitigate data imbalance. Model robustness was assessed using an independent external test set. Visualization of model predictions was facilitated through Gradient-weighted Class Activation Mapping (Grad-CAM) and attention maps generated via Vision Transformer Cross-attention Extension [26].

### Statistical analysis

Statistical analyses were conducted using Python (version 3.10.13) and SPSS (IBM Statistics version 26), with libraries including NumPy, Scikit-learn, SciPy, SHAP, and Statsmodels. For metric data following a normal distribution, t-test was applied, while the Mann-Whitney U test was used for non-normally distributed data. Categorical data were analyzed using the chi-square test. The predictive performance of the model was evaluated using a confusion matrix, with key metrics such as accuracy, sensitivity, specificity, positive predictive value, and negative predictive value. The area under the receiver operating characteristic curve was calculated to assess the model’s overall effectiveness.

## Results

### Data set grouping and allocation

**Figure 2.**
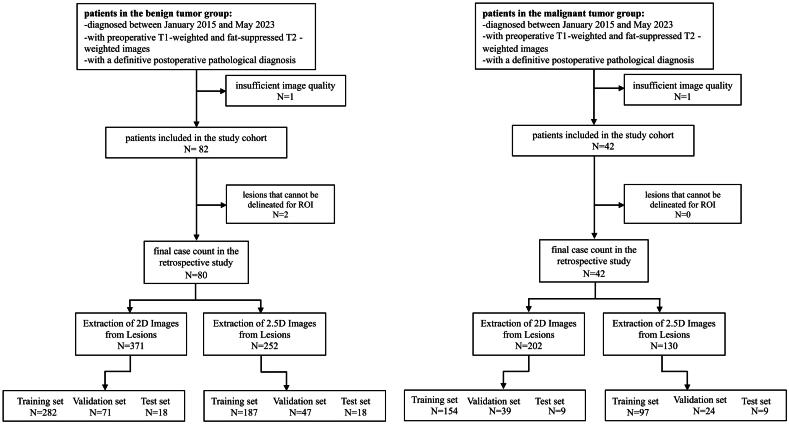
Flowchart of patient selection and slice classification. This figure illustrates the classification process for tumor slices from the T1WI sequence. The T2WI-FS sequence follows the same process.

This study initially included a total of 126 patients, with 99 from Hospital A and 27 from Hospital B. After rigorous screening, 4 cases that did not meet the research criteria were excluded, resulting in a final cohort of 122 patients included in the study (Figure 2). Specifically, the pathological distribution of patients in each group is presented (Table 2). An analysis of the distribution and differences in patient characteristics revealed significant differences in aspects such as shape, size, boundary, invasion of surrounding tissues, lymph node status, and location (Table 3).

**Table 2.** Pathological composition of each group of patients.

	Training set (*n* = 76)	Validation set (*n* = 19)	Test set (*n* = 27)
Benign tumors	50	12	18
Warthin’s tumor	27	5	13
Pleomorphic adenoma	19	7	5
Basal cell adenoma	4	–	–
Malignant tumors	26	7	9
Mucoepidermoid carcinoma	7	1	3
Squamous cell carcinoma	5	1	–
Adenocarcinoma	4	3	–
Lymphoma	3	–	1
Lymphoepithelial carcinoma	1	1	2
Adenoid cystic carcinoma	2	–	2
Salivary duct carcinoma	2	1	1
Acinic cell carcinoma	2	–	–

**Table 3.** Statistical distribution of clinical and imaging features.

Value	Benign tumor	Malignant tumor	Test type	Statistic	*p-*Value
Age (years)	51.96 ± 16.58	48.09 ± 18.67	*T*-test	1.18	0.24
Size (cm)	2.45 ± 0.82	2.87 ± 1.03	*T*-test	−2.48	0.01
Sex			Chi-square	0.423	0.51
Female	31 (0.25)	13 (0.11)			
Male	49 (0.40)	29 (0.24)			
Shape			Chi-square	18.20	0.00
Regular	63 (0.52)	16 (0.13)			
Irregular	17 (0.14)	26 (0.21)			
Boundary			Chi-square	32.55	0.00
Clear	70 (0.58)	15 (0.12)			
Blurred	10 (0.08)	27 (0.22)			
IST			Chi-square	32.55	0.00
Negative	70 (0.58)	15 (0.12)			
Positive	10 (0.08)	27 (0.22)			
Lymph node status			Chi-square	13.23	0.00
Negative	52 (0.43)	12 (0.10)			
Positive	28 (0.23)	30 (0.24)			
Location			Chi-square	20.36	0.00
Superficial lobe	60 (0.49)	14 (0.11)			
Deep lobe	7 (0.06)	12 (0.10)			
Translobar growth	13 (0.11)	16 (0.13)			
Distribution			Chi-square	0.03	0.87
Right side	43 (0.35)	24 (0.20)			
Left side	37 (0.30)	18 (0.15)			

*Note*: IST: invasion of surrounding tissues; lymph node status: ‘negative’ indicates no lymph node enlargement, while ‘positive’ indicates lymph node enlargement. A *p*-value <0.05 denotes a statistically significant difference.

### Clinical model development assessment

**Figure 3.**
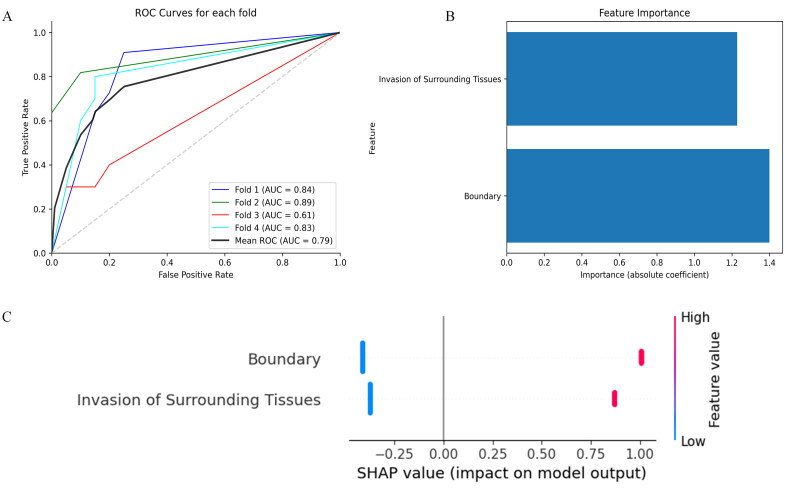
Traditional model performance and feature importance. (A) ROC curves for the logistic regression model across different folds, with AUC values ranging from 0.61 to 0.89, and a mean AUC of 0.79. (B) Feature importance plot showing the weights of invasion of surrounding tissues and boundary in the model. (C) SHAP plot highlighting the impact of boundary and invasion of surrounding tissues on model predictions.

Univariate analysis identified significant differences in tumor size, shape, boundary, invasion of surrounding tissues, lymph node status, and location between benign and malignant groups. Multivariate logistic regression further confirmed that boundary and invasion of surrounding tissues were independent predictors. The logistic regression model built on these independent predictors showed an AUC of 0.79 (95% CI: 0.69–0.90) as shown in Figure 3(A), with a sensitivity of 0.54, specificity of 0.92, accuracy of 0.79, and an F1 score of 0.63. These results indicated that while the model performs reasonably well overall, the sensitivity and F1 score suggested a relatively high rate of missed and false diagnoses in detecting MPGTs. Feature importance analysis revealed (Figure 3(B,C)) that boundary and invasion of surrounding tissues were key contributors to the prediction of malignant tumors.

### DL dataset and training workflow

#### DL model evaluation

**Figure 4.**
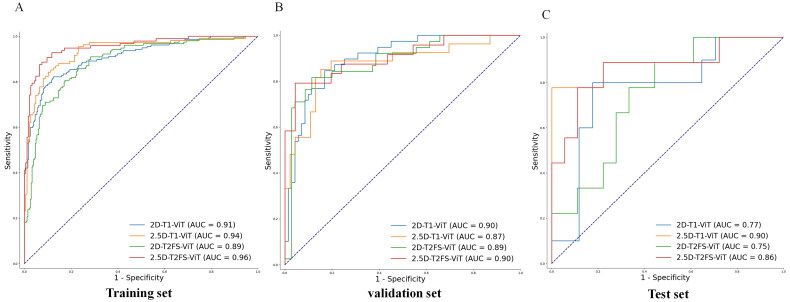
ROC curves of deep learning models based on 2D and 2.5D imaging. ROC curves illustrating the performance of deep learning models based on 2D and 2.5D imaging strategies for the training set (left), validation set (Middle), and test set (right).

The performance metrics for the DL models were summarized in Table 4 and Figure 4. In T1WI-based DL models, the 2.5D model performed slightly lower than the 2D model. In the validation set, the AUC of the 2.5D model was similar to that of the 2D model (AUC of 0.87 vs. 0.90, accuracy of 0.79 vs. 0.80), but the 2.5D model demonstrated superior ability in balancing positive and negative samples (F1 score of 0.76 vs. 0.65). In the test set, although the 2.5D model exhibited better overall diagnostic performance (AUC of 0.90 vs. 0.77), its ability to accurately diagnose benign PGTs based on T1WI was weaker (Specificity: 0.61 vs. 0.76), and the model’s capacity to balance the diagnosis of positive and negative samples also declined (F1 score: 0.67 vs. 0.73). These variations suggested that while the 2.5D strategy based on T1WI showed potential in improving overall AUC and diagnostic performance, it might face challenges in distinguishing between benign and malignant tumors, particularly when dealing with previously unseen external data.

**Table 4.** Evaluation metrics of various DL models.

Model	Set	AUC	95%CI	Accuracy	Sensitivity	Specificity	PPV	NPV	F1score
2D-T1-ViT	Training	0.91	0.85-0.92	0.83	0.66	0.93	0.83	0.83	0.73
	Validation	0.90	0.84-0.95	0.80	0.54	0.94	0.84	0.79	0.65
	Test	0.77	0.56-0.94	0.78	0.80	0.76	0.67	0.87	0.73
2.5D-T1-ViT	Training	0.94	0.93-0.97	0.90	0.85	0.93	0.87	0.92	0.86
	Validation	0.87	0.77-0.96	0.79	0.89	0.74	0.67	0.92	0.76
	Test	0.90	0.70-0.99	0.70	0.89	0.61	0.53	0.92	0.67
2D-T2-ViT	Training	0.89	0.85-0.93	0.83	0.67	0.92	0.83	0.82	0.74
	Validation	0.89	0.82-0.95	0.82	0.55	0.97	0.91	0.81	0.69
	Test	0.75	0.52-0.92	0.67	0.78	0.61	0.50	0.85	0.60
2.5D-T2-ViT	Training	0.96	0.93-0.97	0.91	0.85	0.94	0.87	0.92	0.86
	Validation	0.90	0.81-0.97	0.90	0.79	0.96	0.90	0.90	0.84
	Test	0.86	0.68-0.99	0.81	0.78	0.83	0.70	0.88	0.76

*Note*: AUC: area under the curve; 95% CI: 95% confidence interval; PPV: positive predictive value; NPV: negative predictive value; ViT: vision transformer; 2D-T1-ViT: DL model on 2D T1WI; 2.5D-T1-ViT: DL model on 2.5D T1WI; 2D-T2-ViT: DL model on 2D T2WI-FS, 2.5D-T2-ViT: DL model on 2.5D T2WI-FS.

In the DL models based on T2WI-FS, the 2.5D model demonstrated superior performance compared to the 2D model. In the validation set, the 2.5D model achieved higher AUC and accuracy than the 2D model (0.90 vs. 0.89, 0.90 vs. 0.82) and also exhibited improved capability in balancing positive and negative samples, with an F1 score of 0.84 vs. 0.69. In the test set, the 2.5D model maintained excellent diagnostic performance, with an AUC of 0.86, significantly higher than the 0.75 of the 2D model. Additionally, the 2.5D model showed better accuracy in diagnosing benign parotid gland tumors (BPGTs) compared to the 2D model, with specificity of 0.83 vs. 0.61, while the sensitivity for diagnosing malignant tumors remained the same at 0.78 vs. 0.78. The overall F1 score also remained high for the 2.5D model (0.76 vs. 0.60). These results suggested that the 2.5D strategy based on T2WI-FS not only enhanced overall AUC and diagnostic performance but also improved the model’s ability to distinguish between benign and malignant tumors, even when faced with unseen external data.

**Figure 5.**
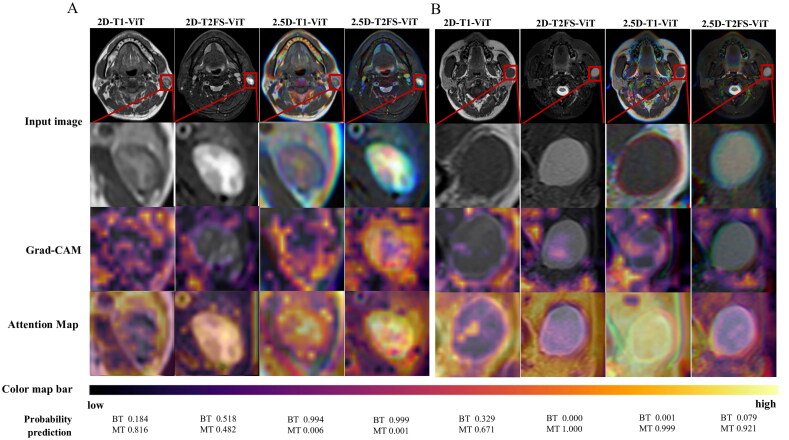
Grad-CAM and attention maps from various DL models. (A) A 43-year-old male with a left-sided Warthin’s tumor confirmed by postoperative pathology. (B) A 26-year-old male with a left parotid mass, diagnosed with low-grade follicular lymphoma after surgery and immunohistochemical evaluation. Grad-CAM analysis shows that for benign tumors, the model focuses on the lesion and its edges, while for malignant tumors, it emphasizes the surrounding tissue, reflecting a more dispersed feature distribution.

Gradient-weighted Class Activation Mapping and attention maps (Figure 5(A,B)) revealed that the model focused on internal and morphological features when predicting BPGTs, while it emphasized features surrounding the tumor for MPGTs.

## Discussion

This study aimed to enhance the diagnostic accuracy of MPGTs using a 2.5D DL strategy that integrated inter-slice information and self-attention mechanisms. The best-performing model, trained using the 2.5D approach, demonstrated excellent diagnostic accuracy for PGTs. On the independent test set, it achieved a strong overall predictive performance (AUC: 0.86) and effectively addressed the higher rates of misdiagnosis and missed diagnoses associated with traditional models in detecting MPGTs (accuracy: 0.81 vs. 0.79, sensitivity: 0.78 vs. 0.54, F1 score: 0.76 vs. 0.63). These results highlighted that incorporating spatial and depth information effectively improved the diagnostic accuracy of PGTs, which was crucial for guiding treatment decisions and improving patient outcomes.

In our conventional clinical model, we employed 4-fold cross-validation to balance the dataset, achieving consistent AUC values (0.78–0.84), which suggested that the model effectively addressed sample imbalance and provided reliable overall performance. However, conventional models demonstrated limited accuracy in diagnosing MPGTs, likely due to the pathological diversity that complicated imaging differentiation. For instance, certain malignancies, such as carcinoma in pleomorphic adenoma and lymphoma, may present imaging characteristics similar to benign tumors, such as clear boundaries and regular shapes. Additionally, reactive lymph node hyperplasia could mimic malignant features, further complicating differentiation. Despite the expectation that malignant tumors typically occurred in the deep lobe, our study observed a higher proportion in the superficial lobe or crossing lobes, possibly reflecting advanced tumor growth by the time of clinical intervention.

Previous DL studies in parotid gland, primarily focusing on 2D convolutional neural networks models, have shown good predictive performance but poor sensitivity, consistent with our findings [27]. To address this, we implemented a 2.5D imaging strategy incorporating inter-slice information and self-attention mechanisms, which successfully improved sensitivity by integrating spatial and depth information during model training. This approach not only enhanced the model’s ability to learn from limited data but also benefited from transfer learning, where pre-trained Vision Transformer models were fine-tuned to recognize specific features of PGTs, crucial for accurate differentiation. Overall, the combination of 2.5D imaging and transfer learning showed potential in improving diagnostic outcomes for rare and complex tumors, although further research was needed to validate these findings in broader and more diverse populations.

This study aimed to mitigate the impact of the rarity and diversity of malignant SGTs on data collection by employing data augmentation techniques. In the final DL models constructed, the 2.5D T2WI-FS model demonstrated superior performance, likely due to the richer lesion signals in T2-weighted images that enhanced feature extraction. This was evident in the model’s higher F1 score and more accurate attention maps compared to T1WI models. However, the decrease in AUC observed in the test set, particularly within the T2WI-FS group, suggested that variability in MRI scanning parameters across institutions could impact model performance, highlighting the need for standardized imaging protocols in clinical practice.

Although radiomics has made progress in differentiating benign tumors from MPGTs [28–30], the lack of standardized procedures limits the consistency and universality of its results. DL offers better repeatability, but research in this field is still evolving, with most studies focusing on 2D models [27,31]. This study confirmed the advantages of using a 2.5D imaging strategy that incorporated inter-slice information for training and generalizing lightweight DL models. To address the rarity of malignant SGTs, we employed data augmentation and whole-tumor slices to expand and balance the dataset. By integrating these with a DL model enhanced by a self-attention mechanism, we achieved a better understanding of lesion information and key features across different tumor layers. This approach significantly improved the diagnostic accuracy for MPGTs.

Fine-Needle Aspiration (FNA) remains a widely utilized diagnostic tool for parotid tumors due to its high sensitivity and specificity in distinguishing benign from malignant lesions [32]. However, the limitations of FNA cannot be overlooked. Its diagnostic accuracy is highly operator-dependent, and factors such as insufficient sampling or the complexity of certain anatomical sites may lead to false-negative results. Additionally, as an invasive procedure, FNA carries potential risks, including infection and localized bleeding. In contrast, the deep learning model developed in this study offers an automated classification approach for parotid tumors, which not only reduces patient discomfort but also eliminates the procedural risks associated with FNA. Preliminary results demonstrate that this method exhibits competitive diagnostic performance, particularly with potential advantages in sensitivity and specificity. Nevertheless, the clinical applicability of this approach requires further validation using larger, multicenter datasets to enhance its robustness and generalizability.

This study has certain limitations. Firstly, its retrospective design may introduce constraints and bias due to reliance on historical data. Secondly, while we employed 2.5D lesion information for model training, the internal details between all lesion layers were not fully captured. Future research will explore DL models based on 3D lesions. Besides, the MRl data were obtained from different devices. Variability in hardware and scanning protocols across devices may introduce heterogeneity in the imaging data, potentially influencing the generalizability of the findings. Future studies should aim to standardize imaging acquisition to minimize such variability. Lastly, the data were sourced from only two centers, limiting the size of the independent test set despite we attempted to expand the training and validation sets. We plan to collect data from additional centers to further validate and optimize the model’s performance.

In conclusion, this study demonstrated that a 2.5D DL model based on MRI, enhanced by inter-slice information and self-attention mechanisms, significantly improved diagnostic accuracy for both BPGTs and MPGTs, particularly in MPGT diagnosis. Although the model’s current utility falls short of FNA, its non-invasive nature and diagnostic potential indicate that, with further refinement and validation, it could become a valuable clinical diagnostic aid.

## Data Availability

The data that support the findings of this study are available from the corresponding author, Xiangning Liu, upon reasonable request.
